# Effect of Different Financial Incentive Structures on Promoting Physical Activity Among Adults: A Randomized Clinical Trial

**DOI:** 10.1001/jamanetworkopen.2019.9863

**Published:** 2019-08-23

**Authors:** Chethan Bachireddy, Andrew Joung, Leslie K. John, Francesca Gino, Bradford Tuckfield, Luca Foschini, Katherine L. Milkman

**Affiliations:** 1 Department of Internal Medicine, Virginia Commonwealth University School of Medicine, Richmond; 2 Leonard Davis Institute Center for Health Incentives and Behavioral Economics, University of Pennsylvania, Philadelphia; 3 Operations, Information and Decisions Department, The Wharton School of the University of Pennsylvania, Philadelphia; 4 Negotiation, Organizations and Markets Unit, Harvard Business School, Boston, Massachusetts; 5 Evidation Health Inc, Santa Barbara, California

## Abstract

**Importance:**

Few adults engage in recommended levels of physical activity. Financial incentives can promote physical activity, but little is known about how the structure of these incentives influences their effectiveness (eg, how incentives are disbursed over time).

**Objective:**

To determine if it is more effective to disburse fixed total financial incentives at a constant, increasing, or decreasing rate to encourage physical activity.

**Design, Setting, and Participants:**

A 2-week randomized clinical trial was conducted from June 2 to 15, 2014, using an online platform that automatically records daily steps of pedometer-wearing users and awards points redeemable for cash. The study population comprised 3515 adult users of the online platform in the lower 70th percentile of steps taken among all users before treatment. Data analyses were performed from August 20, 2014, to February 1, 2018. Analysis was performed on an intent-to-treat basis.

**Interventions:**

Participants were randomized to either a control group or to 1 of 3 intervention groups during the 2 weeks of the study. Participants in the control group received a constant daily rate of $0.00001 per step. The 3 intervention groups received a 20-fold incentive increase ($0.00020 per step) distributed differently during the 2 weeks of the study: at a constant, increasing, or decreasing rate. Reminder emails explaining incentive schedules were sent the day before the intervention and halfway through the 2-week intervention.

**Main Outcomes and Measures:**

Change in mean daily steps during the 2-week intervention and 3 weeks after the intervention. The study had 80% power to detect a difference of 280 steps per day during the intervention at α = .05.

**Results:**

The study included 3515 participants (879 in the control condition, 879 in the constant incentive condition, 881 in the increasing incentive condition, and 876 in the decreasing incentive condition). During the intervention, compared with participants in the control group, participants receiving constant incentives logged 306.7 more steps per day (95% CI, 91.5-521.9 steps; *P* = .005), those receiving decreasing incentives logged 96.9 more steps per day (95% CI, 15.3-178.5 steps; *P* = .02), and those receiving increasing incentives logged no significant change in steps per day (1.5 steps per day; 95% CI, −81.6 to 84.7 steps; *P* = .97). One week after the intervention, compared with participants in the control group, only participants receiving constant incentives logged significantly more steps per day (329.5; 95% CI, 20.6-638.4; *P* = .04). Two and 3 weeks after the intervention, there were no significant differences compared with participants in the control group. Overall, for each $1 spent, participants in the constant incentives group logged 475.4 more steps than those in the increasing incentives group and 429.3 more steps than those in the decreasing incentives group.

**Conclusions and Relevance:**

This study found that financial incentives for physical activity were more effective during a payment period when they were offered at a constant rate rather than an increasing or decreasing rate. However, this effectiveness dissipated shortly after the incentives were removed.

**Trial Registration:**

ClinicalTrials.gov identifier: NCT02154256

## Key Points

**Question:**

Is it more effective to disburse fixed total financial incentives at a constant, increasing, or decreasing rate to encourage physical activity?

**Findings:**

In this randomized clinical trial, financial incentives for physical activity were significantly more effective at motivating activity during and immediately after a payment period if they were offered at a constant rate rather than an increasing or decreasing rate.

**Meaning:**

This finding has implications for the design and delivery of incentive programs to promote healthy behaviors.

## Teaser

This randomized clinical trial examines whether it is more effective to disburse fixed total financial incentives at a constant, increasing, or decreasing rate to encourage physical activity among pedometer-wearing adults.

## Introduction

Physical inactivity has been implicated as a major risk factor for disability and death globally, on par with obesity and smoking, yet it receives considerably less attention than other risk factors.^1,2^ Inactivity accounts for 9% of premature mortality.^2^ In the United States, inactive individuals older than 50 years would gain 1.3 to 3.7 years of life expectancy if they became active.^3^ Activity alone can reduce the risk of developing diabetes, cardiovascular disease, and colon and breast cancers and improve bone and mental health; however, less than half of adults in the United States engage in recommended levels of physical activity.^4^ The benefits of activity and the costs of inactivity are clear, but motivating individuals to increase their activity is challenging.

Financial rewards are a useful tool for encouraging healthy behaviors, including smoking cessation, eating nutritious foods, physical activity, and weight loss.^5,6,7,8,9,10,11,12,13,14^ At least 15 state Medicaid programs and more than one-quarter of large employers offer financial incentive–based health and wellness programs.^15,16^

Recent literature suggests that the principles of behavioral economics can be effectively harnessed to design and deliver incentives capable of changing health behaviors such as physical activity.^17^ We understand that rewards conditioned on performing specific behaviors are more likely to be successful in promoting exercise, for instance, compared with unconditional rewards encouraging behavior change (such as free gym memberships). In a recent systematic review, Mitchell et al^18^ identified key principles of effective incentive design to promote physical activity, including immediate incentives, realistic daily goals, and longer interventions (>24 weeks) among less-active adults. To have an effect, the size of the incentive does not have to be large (approximately $1 per day), and its effect can be multiplied through frequent and personalized feedback.

Still, much remains unknown. First, although we understand that small daily incentives over time can be effective, few studies have sought to assess how those small daily incentives should be distributed over time. A critical question is therefore how to disburse incentives for maximal effect. This is the primary question of the present research. Second, although financial incentives can encourage healthy behaviors, including exercise, it is unclear how to create behavior change that is sustained after incentives are inevitably removed. Among the studies demonstrating the benefits of financial incentives, few have measured postintervention behavior, and fewer still have demonstrated evidence of behavior change lasting beyond the period when incentives were offered.^7,8,9,17,18^

Although maintaining the same financial incentive over time has the benefit of simplicity, it may not be the best way to foster sustained behavior change. Starting with a small incentive and increasing it over time may help individuals gradually build a habit by preventing the development of tolerance to a specific incentive value, just as patients may develop tolerance to medications and require an increased dosage to maintain the same effect.^19^ However, starting with a large incentive may help motivate individuals to overcome inertia and initiate a new routine.^20,21^ Gradually reducing incentives over time from an initially high level may then help diminish individuals’ reliance on financial rewards for motivation to exercise, making it easier to transition to nonincentivized engagement.^22,23,24^

Our primary objective was to compare 3 different 2-week incentive programs with rewards for daily steps taken to determine which was the most effective for increasing the number of steps both during and after the intervention. We build on the existing literature by leveraging an online platform with points-based daily financial incentives enhanced with frequent, personalized feedback to conduct this study. Each of the 3 programs offered the same total incentives, which were distributed differently: increasing, decreasing, or constant over time. In a 4-group randomized clinical trial, we compared the effectiveness of these incentive programs vs a control group.

## Methods

### Study Design

We conducted a field experiment with Achievement (formerly called AchieveMint),^25^ an online platform owned by Evidation Health that automatically records the daily steps of users who connect their pedometers to the platform and awards them points redeemable for cash. One step earns $0.00001 (ie, 10 000 steps = $0.10). We tested whether offering incentives to users that are 20 times as large as usual during 2 consecutive weeks would change the steps taken during and after the intervention compared with a control group, and we tested the size of the change in the number of steps. Our clinical trial preregistration was vague. The purpose of the project was to examine the effect of financial incentives on the achievement of physical activity goals. We limited the follow-up analysis to 3 weeks after the intervention because the intervention lasted for only 2 weeks, and the effects dissipated after an additional 2 weeks. We determined that a lengthier follow-up analysis than the one we present would not add value. This study was approved by the University of Pennsylvania institutional review board, and a waiver of informed consent was granted because personal identifiers were removed from the data. The trial protocol is available in Supplement 1.^1^ This study followed the Consolidated Standards of Reporting Trials (CONSORT) reporting guidelines.

### Setting and Participants

Participants were adult users of the online platform who logged steps using a pedometer at least once between May 9 and 22, 2014 (the date that participant selection occurred). At the time of the study, the online platform did not routinely collect demographic information on its users; therefore, we do not have demographic data on study participants.

To maximize the health effect of our intervention, we excluded the most active users and conducted our study among users whose logged steps were in the bottom 70th percentile of all users between May 9 and 22, 2014. We calculated that a sample of 3515 participants would allow us to detect a difference of 280 steps per day at α = .05 with 80% power. Based on the resources available and the study power calculations, we decided that a 2-week intervention would be sufficient to answer the study’s key question regarding which financial incentive structure would most effectively promote physical activity.

### Randomization and Interventions

**Figure 1.**
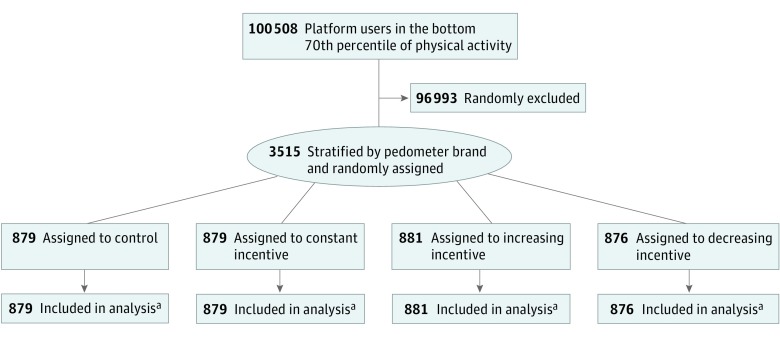
Study Flow Diagram ^a^Data on step count were captured through and contingent on daily pedometer use, which varied between 82.8% and 84.2% among conditions before the intervention and 76.4% to 79.7% during the intervention. To account for missing data, we used an intent-to-treat approach in which we replaced missing pedometer data with a participant’s preintervention daily step count of more than 2000 steps stratified by day of week.

**Figure 2.**
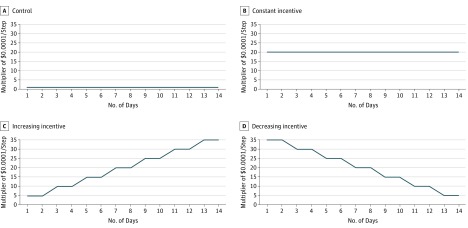
Relative Incentive Rates Offered per Step Incentive rate on each of 14 days of intervention by experimental condition, where the y-axis represents a multiplier of the control incentive rate ($0.00001/step). For example, in panel B, the constant incentive condition has a multiplier of 20 for each of 14 days, and so the daily incentive rate would be 20 × $0.00001/step = $0.00020/step.

Participants were stratified by 1 of 9 pedometer brands (ActiveBeats, BodyMedia FIT, FitBug, Fitbit, Jawbone UP, MapMyWalk, Misfit Wearables, Moves, and Withings) and randomly assigned to 1 of 4 experimental conditions as outlined in Figure 1 and Figure 2: a control condition (in which participants received incentives as usual: $0.00001 per step [ie, $0.10 per 10 000 steps]) or 1 of 3 treatment conditions. In the 3 treatment conditions, participants were offered a mean of 20 times their usual points per step (ie, $2.00 per 10 000 steps) during the 2-week intervention period. Thus, comparing the control condition with these 3 treatment conditions enabled us to test the effect of a 20-fold incentive increase on walking behavior. Comparing the 3 treatment conditions enabled us to test the effect of incentive structure, our primary interest.

In the constant incentive condition, participants were offered $0.00020 per step every day. In the increasing incentive condition, they were initially offered $0.00005 per step (ie, $0.50 per 10 000 steps); this amount increased by $0.00005 per step every 2 days up to a maximum of $0.00035 per step (ie, $3.50 per 10 000 steps) on the last 2 days. In the decreasing incentive condition, participants were initially offered $0.00035 per step; this decreased by $0.00005 per step every 2 days down to a minimum of $0.00005 per step on the last 2 days. The schedule of incentives is detailed further in the eTable in Supplement 2.

Routinely, users of the online platform receive an update email on Sunday reflecting their weekly earnings in points and dollars. During the 2-week intervention, these Sunday emails continued to be sent. Study-specific emails were also sent. The day before the intervention began (Sunday, June 1, 2014), all study participants received an email describing the program designed to help them increase their physical activity (eFigures 1, 2, 3, and 4 in Supplement 2). In the treatment groups, participants received a precise schedule detailing the incentives that they would receive for each step taken on each day during the subsequent 2 weeks. Data on all study participants who wore pedometers on a given day and synchronized their pedometers with the online platform within 7 days were recorded in our data set as steps taken; otherwise, data were recorded as missing observations, allowing for analyses accounting for missing observations in a variety of ways. On day 7 of the 14-day intervention, all study participants received a reminder email encouraging them to be physically active (eFigure 5 and eFigure 6 in Supplement 2). The reminder email for treatment participants also included their specific incentive rate.

### Outcomes

We report individual daily step counts for 8 weeks total: 3 weeks before the intervention, 2 weeks during the intervention, and 3 weeks after the intervention. The primary outcome measure was change in daily steps taken, which was collected remotely through participants’ pedometers. The intervention began on June 2, 2014, and concluded on June 15, 2014. Initial data analysis occurred in 2014. Based on peer feedback, a sensitivity analysis was conducted, and all analyses were finalized in 2018. Data analyses were performed from August 20, 2014, to February 1, 2018.

### Statistical Analysis

Prior studies have demonstrated that daily step counts measured by pedometer that are lower than 2000 are unlikely to be reflective of true daily step count values; we define a missing data day as any day with fewer than 2000 recorded steps.^26^ To address the possibility that some participants walked without pedometers, we present all analyses in 2 different ways (the results of which converge on the same conclusion).

In our primary analysis, we used an intent-to-treat strategy in which we replaced missing data with a mean of a given participant’s preintervention daily step counts greater than 2000 steps, stratified by day of week to account for person-within-week differences in physical activity (ie, a participant may routinely get more physical activity on Saturdays than on Wednesdays). To further minimize the potential for bias, we conducted a sensitivity analysis in which we deleted all daily step data recording fewer than 2000 steps—an approach that would bias toward a null effect.

We used ordinary least squares regression to determine the overall and separate effects of our 3 treatment groups (constant, increasing, and decreasing rates) on participants’ daily steps. We included person-by-day-of-week fixed effects and cluster SEs by person-by-day-of-week to control for individual differences in steps and further for differences in participant routines that vary by day of week; these fixed effects also capture condition assignment. In addition, we included fixed effects by pedometer brand and for each day of the year to account for seasonal conditions that may influence step count. We used Wald tests to assess differences between treatment conditions and conducted a cost-effectiveness analysis of additional steps taken per $1 paid to each treatment condition participant relative to control participant. All *P* values were from 2-sided tests and results were deemed statistically significant at *P* < .05. Stata version 15 was used for analyses (StataCorp).

## Results

The sample of 3515 participants was distributed randomly among the control (n = 879), constant incentive (n = 879), increasing incentive (n = 881), and decreasing incentive (n = 876) conditions. In the 3 weeks before the intervention, the mean (SD) number of daily steps across all study participants was 6804.5 (3506.9). Before the intervention, each day, on average, 15 391 of 18 459 participants in the control group (83.4%), 15 317 of 18 501 in the increasing incentive condition (82.8%), 15 497 of 18396 in the decreasing incentive condition (84.2%), and 15 527 of 18 459 in the constant incentive condition (84.1%) used their pedometers. During the intervention, each day, on average, 9398 of 12 306 participants in the control group (76.4%), 9747 of 12 334 in the increasing incentive condition (79.0%), 9701 of 12 264 in the decreasing incentive condition (79.1%), and 9804 of 12 306 in the constant incentive condition (79.7%) used their pedometers. Although there were significant differences in pedometer adherence between conditions in the preintervention and intervention periods, the differences were small. To address this difference and the possibility that some participants walked without pedometers, we used an intent-to-treat approach in which we replaced missing data.

### Effect of a 20-Fold Increase in Incentives

Among the 3 treatment groups combined, participants took an estimated 135.0 additional daily steps (95% CI, 41.0-228.9 steps) relative to the participants in the control group during the intervention period (*P* = .005). In the 3 weeks after the intervention, there were no significant differences.

**Figure 3.**
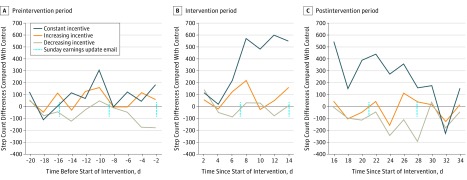
Unadjusted Mean Differences in Step Count Compared With Control by Experimental Condition

Figure 3 shows the unadjusted differences in mean steps taken by treatment participants compared with control participants for 3 weeks before, 2 weeks during, and 3 weeks after the intervention. Treatment participants experienced an increase in physical activity midway through the intervention (when the regular Sunday earnings update email was sent), and the increase was particularly large for those in the constant incentive condition. In the more conservative sensitivity analysis in which we deleted all step count data less than 2000, we found qualitatively similar results.

### Effect of Incentive Structure

#### During the Intervention

Table 1 presents the results of regressions and Wald tests comparing the effectiveness of each treatment group relative to the control group and relative to each other during the 2-week intervention. Participants in the constant incentive condition logged 306.7 additional daily steps (95% CI, 91.5-521.9 steps) relative to those in the control condition (*P* = .005), 305.1 additional daily steps (95% CI, 89-521.2 steps) relative to those in the increasing incentive condition (*P* = .006), and 209.8 additional daily steps (95% CI, −5.7 to 425.3 steps) relative to those in the decreasing incentive condition (*P* = .06). Participants in the decreasing incentive condition demonstrated a small increase in daily steps relative to those in the control condition (96.9 additional daily steps; 95% CI, 15.3-178.5 steps; *P* = .02) and relative to those in the increasing incentive condition (95.3 additional daily steps; 95% CI, 11.3-179.3 steps; *P* = .03). Participants in the increasing incentive condition did not log significantly more steps per day than those in the control condition (1.5; 95% CI, −81.6 to 84.7; *P* = .97). In the sensitivity analysis, we found similar results, except there was no longer a statistically significant effect of decreasing incentives compared with control during the intervention period (80.5 steps; 95% CI, −38.5 to 199.4 steps; *P* = .19).

**Table 1.** Adjusted Mean Daily Step Counts by Experimental Condition During the 14-Day Intervention Period^a^

Characteristic	Step Count (95% CI)							
	Main Model^b^				Sensitivity Analysis^c^			
	Control	Constant Incentive Condition	Increasing Incentive Condition	Decreasing Incentive Condition	Control	Constant Incentive Condition	Increasing Incentive Condition	Decreasing Incentive Condition
Daily step count	6968.9 (6912.4 to 7025.4)	7275.5 (7075.5 to 7475.6)	6970.4 (6910.6 to 7030.2)	7065.8 (7008.1 to 7123.4)	7386.1 (7301.7 to 7470.6)	7803.5 (7498.8 to 8108.1)	7389.4 (7303.7 to 7475.2)	7466.6 (7384.3 to 7548.9)
Difference in daily step count								
Relative to control	NA	306.7 (91.5 to 521.9)^d^	1.5 (−81.6 to 84.7)	96.9 (15.3 to 178.5)^e^	NA	417.3 (91.7 to 742.9)^e^	3.3 (−118.1 to 124.7)	80.5 (−38.5 to 199.4)
Relative to increasing incentive condition	NA	305.1 (89.0 to 521.2)^d^	NA	NA	NA	414.0 (88.4 to 739.6)^e^	NA	NA
Relative to decreasing incentive condition	NA	NA	−95.3 (−179.3 to −11.3)^e^	NA	NA	NA	−77.1 (−197.0 to 42.8)	NA
Relative to constant incentive condition	NA	NA	NA	−209.8 (−425.3 to 5.7)^f^	NA	NA	NA	−336.9 (−661.6 to −12.2)^e^

Abbreviation: NA, not applicable.

^a^ Ordinary least square regression models used to generate these estimated step counts include fixed effects for person-by-day-of-week, day-of-year, and pedometer brand. Robust SEs are clustered by participant-day-of-week. Between intervention group differences were calculated using Wald tests.

^b^ The main model uses an intent-to-treat strategy and replaces missing data based on a mean of preintervention step counts of more than 2000 steps, stratified by day of week, which would bias the findings slightly toward a null effect (N = 815 480; *R*^2^ = 0.38).

^c^ The sensitivity analysis uses an intent-to-treat strategy and includes only step count data of 2000 steps or more, stratified by day of week, which would bias the findings more heavily toward a null effect (N = 509 275; *R*^2^ = 0.26).

^d^ *P* < .01.

^e^ *P* < .05.

^f^ *P* < .10.

#### After the Intervention

Table 2 presents the effectiveness of each treatment group in the 3 weeks after the intervention. In the first week after the intervention, participants in the constant incentive condition took 329.5 more daily steps (95% CI, 20.6-638.4 steps) than those in the control condition (*P* = .04), 397.8 more daily steps (95% CI, 89.2-706.4 steps) than those in the increasing incentive condition (*P* = .01), and 308.6 more daily steps (95% CI, 0.1-617.1 steps) than those in the decreasing incentive condition (*P* = .05). There were no significant differences between the those in the increasing incentive condition and those in the control condition (−68.3 steps; 95% CI, −174.6 to 38.1 steps; *P* = .21) and between those in the decreasing incentive condition and those in the control condition (21.0 steps; 95% CI, −84.9 to 126.8 steps; *P* = .70).

**Table 2.** Adjusted Mean Daily Step Counts by Experimental Condition During the 21-Day Postintervention Period^a^

Characteristic	Step Count (95% CI)							
	Main Model^b^				Sensitivity Analysis^c^			
	Control	Constant Incentive Condition	Increasing Incentive Condition	Decreasing Incentive Condition	Control	Constant Incentive Condition	Increasing Incentive Condition	Decreasing Incentive Condition
1 wk after intervention (days 1-7)								
Daily step count	6985.8 (6910.7 to 7061.0)	7315.4 (7023.4 to 7607.3)	6917.6 (6843.4 to 6991.8)	7006.8 (6933.3 to 7080.4)	7426.6 (7304.5 to 7548.8)	7912.0 (7434.0 to 8390.0)	7304.6 (7189.4 to 7419.8)	7396.6 (7282.6 to 7510.7)
Difference in daily step count								
Relative to control group	NA	329.5 (20.6 to 638.4)^d^	−68.3 (−174.6 to 38.1)	21.0 (−84.9 to 126.8)	NA	485.4 (−20.1 to 990.9)^e^	−122.0 (−290.2 to 46.2)	−30.0 (−197.4 to 137.5)
Relative to increasing incentive condition	NA	397.8 (89.2 to 706.4)^d^	NA	NA	NA	607.4 (103.7 to 1111.1)^d^	NA	NA
Relative to decreasing incentive condition	NA	NA	−89.2 (−194.4 to 16.0)^e^	NA	NA	NA	−92.0 (−254.5 to 70.5)	NA
Relative to constant incentive condition	NA	NA	NA	−308.6 (−617.1 to −0.1)^d^	NA	NA	NA	−515.4 (−1018.8 to −12.0)^d^
2 wk after intervention (days 8-14)								
Daily step count	7025.9 (6952.3 to 7099.6)	7239.4 (6947.7 to 7531.2)	6924.2 (6846.9 to 7001.6)	6942.3 (6869.6 to 7015.1)	7472.3 (7354.6 to 7590.1)	7791.4 (7319.1 to 8263.7)	7288.9 (7169.4 to 7408.4)	7259.5 (7147.0 to 7372.0)
Difference in daily step count								
Relative to control group	NA	213.5 (−94.8 to 521.8)	−101.7 (−209.2 to 5.8)^e^	−83.6 (−187.7 to 20.6)	NA	319.1 (−179.7 to 817.9)	−183.4 (−351.5 to −15.3)^d^	−212.9 (−376.2 to −49.6)^d^
Relative to increasing incentive condition	NA	315.2 (6.0 to 624.4)^d^	NA	NA	NA	502.5 (3.5 to 1001.5)^d^	NA	NA
Relative to decreasing incentive condition	NA	NA	−18.1 (−125.0 to 88.8)	NA	NA	NA	29.4 (−135.1 to 193.9)	NA
Relative to constant incentive condition	NA	NA	NA	−297.1 (−605.1 to 10.9)^e^	NA	NA	NA	−532.0 (−1029.4 to −34.6)^d^
3 wk after intervention (days 15-21)								
Daily step count	6981.7 (6903.3 to 7060.1)	6959.0 (6829.0 to 7088.9)	6905.4 (6828.0 to 6982.8)	7041.7 (6970.1 to 7113.2)	7423.0 (7291.8 to 7554.3)	7350.7 (7131.2 to 7570.3)	7268.6 (7143.1 to 7394.0)	7406.8 (7290.8 to 7522.8)
Difference in daily step count								
Relative to control group	NA	−22.8 (−177.3 to 131.8)	−76.3 (−187.1 to 34.5)	59.9 (−46.8 to 166.7)	NA	−72.3 (−333.6 to 188.9)	−154.5 (−336.4 to 27.4)^e^	−16.2 (−191.7 to 159.2)
Relative to increasing incentive condition	NA	53.6 (−100.5 to 207.7)	NA	NA	NA	82.1 (−176.4 to 340.6)	NA	NA
Relative to decreasing incentive condition	NA	NA	−136.3 (−242.3 to −30.3)^d^	NA	NA	NA	−138.2 (−309.3 to 32.9)	NA
Relative to constant incentive condition	NA	NA	NA	82.7 (−68.4 to 233.8)	NA	NA	NA	56.1 (−197.5 to 309.7)

Abbreviation: NA, not applicable.

^a^ Ordinary least square regression models used to generate these estimated step counts include fixed effects for person-by-day-of-week, day-of-year, and pedometer brand. Robust SEs are clustered by participant-day-of-week. Between-intervention group differences were calculated using Wald tests.

^b^ The main model uses an intent-to-treat strategy and replaces missing data based on a mean of preintervention step counts of more than 2000 steps, stratified by day of week, which would bias the findings slightly toward a null effect (N = 815 480; *R*^2^ = 0.38).

^c^ The sensitivity analysis uses an intent-to-treat strategy and only includes step count data of 2000 steps or more, stratified by day of week, which would bias the findings more heavily toward a null effect (N = 509 275; *R*^2^ = 0.26).

^d^ *P* < .05.

^e^ *P* < .10.

In the second week after the intervention, participants in the constant incentive condition logged significantly more daily steps than those in the increasing incentive condition (315.2 steps; 95% CI, 6.0-624.4 steps; *P* = .046). Participants in the constant incentive condition also logged more daily steps than those in the control and decreasing incentive conditions, but these differences were not significant (control group: 213.5 steps; 95% CI, −94.8 to 521.8 steps; *P* = .18; decreasing incentive condition: 297.1 steps; 95% CI, −10.9 to 605.1 steps; *P* = .06). There were no significant differences between the increasing and decreasing incentive conditions and the control condition (increasing incentive condition: −101.7 steps; 95% CI, −209.2 to 5.8 steps; *P* = .06; decreasing incentive condition; −83.6 steps; 95% CI, −187.7 to 20.6 steps; *P* = .12).

In the third week after the intervention, there were no significant differences in steps taken between the constant incentive condition and the increasing incentive condition (53.6 steps; 95% CI, −100.5 to 207.7 steps; *P* = .77), the decreasing incentive condition (−82.7 steps; 95% CI, −233.8 to 68.4 steps; *P* = .18), or the control condition (−22.8 steps; 95% CI, −177.3 to 131.8 steps; *P* = .27). There was, however, a significant increase of 136.3 daily steps (95% CI, 30.3-242.3 steps) in the decreasing incentive condition compared with the increasing incentive condition (*P* = .01).

In the sensitivity analysis, we found similar results, except that at 1 week after the intervention, there was no longer a statistically significant effect of constant incentives compared with control (485.4 steps; 95% CI, −20.1 to 990.9 steps; *P* = .06). Constant incentives demonstrated a sustained effect 1 week after the intervention compared with the increasing incentive condition (607.4 steps; 95% CI, 103.7-1111.1 steps; *P* = .01) and decreasing incentive condition (515.4 steps; 95% CI, 12.0-1018.8 steps; *P* = .04). There was no statistically significant difference in steps 1 week after the intervention between the constant incentive and control conditions (485.4 steps; 95% CI, −20.1 to 990.0 steps; *P* = .06). Two weeks after the intervention, there was a statistically significant effect of increasing incentives compared with the control groups (−183.4 steps; 95% CI, −351.5 to −15.3 steps; *P* = .03), decreasing incentives compared with the control group (−212.9 steps; 95% CI, −376.2 to −49.6 steps; *P* = .01), and decreasing incentives compared with constant incentives (−532.0 steps; 95% CI, −1029.4 to −34.6 steps; *P* = .04). Three weeks after the intervention, there was no longer a statistically significant effect of increasing incentives compared with decreasing incentives (−138.2 steps; 95% CI, −309.3 to 32.9 steps; *P* = .11).

### Cost-effectiveness

During the intervention, participants in the constant incentive condition were paid a mean of $15.48 per person compared with a mean of $14.54 per person in the increasing incentive condition and a mean of $14.67 per person in the decreasing incentive condition. Compared with the control group and including postintervention effects, for each additional $1 paid, there were 582.4 additional steps per participant in the constant incentive condition, 107.0 additional steps per participant in the increasing incentive condition, and 153.1 additional steps per participant in the decreasing incentive condition.

## Discussion

 To our knowledge, this is one of the largest randomized clinical trials of financial incentives for physical activity. We tested the short-term effect of different financial incentive structures on physical activity. Incentive structure affected physical activity during the 2-week intervention; the constant incentives significantly increased physical activity relative to all other conditions—control, increasing incentives, and decreasing incentives. These effects held for 1 week after the incentives had been removed. These effects dissipated 2 to 3 weeks after the intervention. Similar to prior studies, after the withdrawal of incentives, physical activity tapered in all conditions.^14,27^

We conclude that incentive structure—independent from incentive size, which was the same across our treatment groups—affects physical activity at least during the period when incentives are offered. Thus, in designing wellness programs, incentive designers and policy makers should consider not simply the magnitude of incentives, but also their structure.^6,11^

The control group effectively received an incentive at a constant rate, just 20-fold lower than the incentive in the treatment conditions. The constant incentive rate structure was so effective that, during the intervention, the control group performed equally as well as those receiving a 20-fold greater incentive delivered at an increasing rate and only marginally worse than those receiving a 20-fold greater incentive delivered at a decreasing rate.

Our results on the comparative effectiveness of constant vs decreasing incentives are consistent with findings from Carrera et al^27^ directly comparing the association of a constant incentive and a decreasing incentive with gym initiation and attendance for 8 weeks among employees of a Fortune 500 company. They found that among nongym members, the constant and decreasing incentives were equally effective in increasing gym join rates. However, among existing gym members, the constant incentive was significantly more effective than the decreasing incentive in motivating physical activity during and after the intervention. Their findings complement a host of studies exploring different payment disbursement schemes for motivating physical activity.^8,9,10,11,27,28,29^

Only a handful of studies on financial incentives for exercise and physical activity have measured and demonstrated behavior change after the intervention.^8,9,10,27,30^ These studies differ from our study in a number of ways: almost all the studies incentivized and measured gym attendance rather than step count, lasted 4 weeks or longer, provided an incentive with a daily expected value more than twice that of our study ($1.40), and recruited samples of fewer than 1000 participants.^8,9,10,27,30^

Our findings raise the question of why incentives delivered at a constant rate were more effective than other incentive disbursement strategies. One potential explanation is that the constant incentive was easier to remember and therefore more salient and effective at promoting physical activity.^31^ By contrast, in the other disbursement strategies, getting paid different amounts for doing the same activity may have been confusing, or even felt unfair, potentially contributing to the relative ineffectiveness of those strategies.^32^ Further research exploring these and other possibilities would be valuable.

Prior work suggests a differential and often lesser effect of financial incentives among those with existing exercise habits.^9,27^ Users of the online platform that we studied have higher daily step counts than the average US adult, which is why, for our study, we sampled from users in the bottom 70% of physical activity. As a result, our study findings reflect a population with similar baseline physical activity as the US population.^33^ However, we cannot say as much about how our incentive conditions might affect those who are on the extremes of physical activity, including those who are sedentary.

### Limitations

This study has several limitations. First, we were dependent on participants’ device-wearing behaviors. We could not detect steps if a participant did not wear the pedometer, resulting in missing data. Missing data are a common challenge when conducting experimental research in real-world settings. Prior studies have dealt with missing or partially recorded step data by excluding or replacing the data with a uniform step number. These approaches have their own shortcomings because deleting the data biases the findings toward a null effect and replacing missing data with zeros biases the findings toward finding an effect because of better observability in treatment groups (who are more incentivized to wear pedometers). Instead, as described in the Methods section, we took a more conservative approach, replacing missing data with the mean of preintervention steps greater than 2000 and used an intent-to-treat analytic strategy. This approach has a slight bias toward a null effect but is more balanced than prior approaches to the common occurrence of missing step data. Furthermore, all analyses are presented using an even more conservative approach of deleting all step data below a certain threshold, consistent with prior research.^29^

Second, pedometers restricted us to step count, even though other metrics such as metabolic equivalents or minutes of moderate to vigorous physical activity might be more relevant to long-term health outcomes. Third, despite randomization, preintervention mean daily steps were significantly higher among participants in the increasing incentive condition compared with those in the decreasing incentive condition. We attempted to minimize this bias through a focus on change in mean daily steps and inclusion of fixed effects to account for time-invariant differences among participants. More important, this limitation does not apply to comparisons with the constant condition because there were no significant differences in preintervention mean daily steps between the constant and increasing, decreasing, or control incentive conditions.

Fourth, we do not have demographic data for the population, which may have revealed insights and further strengthened our regression analyses. We attempt to address this limitation through an advanced analytic approach that includes fixed effects by person-day-of-week, pedometer, and day-of-year and clustered SEs by person-day-of-week.

Fifth, compared with prior experiments on incentives for health behaviors, our intervention period of 2 weeks was relatively short, and our incentive was relatively small. On the other hand, the incentives, in particular the constant ones, had an effect despite their size. Incentives delivered for a longer period may lead to greater behavior change during and after an intervention.^7,8,9,10,27^

Sixth, the study was not well powered to detect differences in step count long after the intervention. Nonetheless, we found that participants in the constant incentive condition logged significantly more steps compared with those in the increasing incentive condition in the two weeks after the intervention. Although this experiment was designed to assess which incentive condition produced the most physical activity during and briefly after the intervention, it cannot answer another important and broader question, which is what incentive structure is optimal to promote long-term changes in physical activity.

## Conclusions

To our knowledge, this is one of the largest randomized clinical trials of financial incentives for physical activity. For the same possible total earnings, daily incentives of constant value delivered for 2 weeks were more effective in promoting physical activity compared with incentives of increasing or decreasing value. These findings have implications for the psychology of behavior change and suggest that incentive structure should be a key design consideration in the delivery of health incentive programs. Future research should continue to explore strategies to improve health through incentives and remote technology, with an eye toward building persistent behaviors that lead to habit formation.

## Notes

Supplement 1. **Trial Protocol**

Supplement 2. **eTable.** Schedule of Incentive Rates Offered per Step on Each of 14 Days of Intervention by Experimental Condition **eFigure 1.** Program Announcement Email for the Control Condition **eFigure 2.** Program Announcement Email for the Constant Incentive Condition **eFigure 3.** Program Announcement Email for the Increasing Incentive Condition **eFigure 4.** Program Announcement Email for the Decreasing Incentive Condition **eFigure 5.** Reminder Email for the Control Condition **eFigure 6.** Reminder Email for the Treatment Conditions

Supplement 3. **Data Sharing Statement**
