# Supplementary material for: Effect of Different Financial Incentive Structures on Promoting Physical Activity Among Adults: A Randomized Clinical Trial
Source: JAMA Netw Open. 2019 Aug 23;2(8):e199863. doi: 10.1001/jamanetworkopen.2019.9863 (PMC6714021; doi:10.1001/jamanetworkopen.2019.9863)
Supplement: Supplement 1. — Trial Protocol [file jamanetwopen-2-e199863-s001.pdf]

Modification

Basic Info

Confirmation Number:	**bejffgcf**
Protocol Number:	**819369**
Created By:	**PENG, LILI**
Principal Investigator:	**MILKMAN, KATHERINE L**
Protocol Title:	**AchieveMint: Accomplishing Goals and Forming Habits**
Short Title:	**AchieveMint: Goals and Habits**
Protocol Description:	**The goal of this set of projects is to apply insights from behavioral economics literature and theory to help people accomplish personal goals and form good personal habits. We will be working with a company called AchieveMint, a company that has developed an application and software process to encourage users' healthy activities and develop insights into their routines.**
Submission Type:	**Social and Biological Sciences**
Application Type:	**PRIME**

PennERA Protocol Status

Approved

Resubmission*
No

Are you submitting a Modification to this protocol?*

Yes

Current Status of Study

Study Status

Study has not begun (no subjects entered)

If study is currently in progress, please enter the following

Number of subjects enrolled at Penn since the study was initiated

0

Actual enrollment at participating centers

0

If study is closed to further enrollment, please enter the following

Number of subjects in therapy or intervention

0

Number of subjects in long-term follow-up only

0

IRB Determination
If the change represents more than minimal risk to subjects, it must be reviewed and approved by the IRB at a convened meeting. For a modification to be considered more than minimal risk, the proposed change would increase the risk of discomfort or decrease benefit. The IRB must review and approve the proposed change at a convened meeting before the change can be implemented unless the change is necessary to eliminate an immediate hazard to the research participants. In the case of a change implemented to eliminate an immediate hazard to participants, the IRB will review the change to determine that it is consistent with ensuring the participant′s continued welfare. Examples: Convened Board Increase in target enrollment for investigator initiated research or potential Phase I research Expanding inclusion or removing exclusion criteria where the new population may be at increased risk Revised risk information with active participants Minor risk revisions that may affect a subject′s willingness to continue to participate Expedited Review Increase in target enrollment at Penn where overall enrollment target is not exceeded or potentially sponsored research Expanding inclusion or removing exclusion where the new population has the same expected risk as the previous, based on similarities of condition Revised risk information with subjects in long-term follow-up Minor risk revisions with no subjects enrolled to date
Expedited Review

Modification Summary
Please describe any required modification to the protocol. If you are using this form to submit an exception or report a deviation, enter 'N/A' in the box below.
We are adding an appendix to the protocol that indicates a study that we plan to run.

Risk / Benefit
Does this amendment alter the Risk/Benefit profile of the study?
No

Change in Consent
Has there been a change in the consent documents?
No

If YES, please choose from the options below regarding re-consenting

Deviations

Are you reporting a deviation to this protocol?*

No

Exceptions

Are you reporting an exception to this protocol?*

No

Protocol Details

Resubmission*
Yes

Study Personnel

Principal Investigator

Name:	**MILKMAN, KATHERINE L**
Dept / School / Div:	**709 - Operations, Information and Decisions**
Campus Address Mail Code	**6340**
Address:	**HUNTSMAN HALL<br>3730 WALNUT ST**
City State Zip:	**PHILADELPHIA PA 19104-6340**
Phone:	**215-898-5873**
Fax:	**-**
Pager:	
Email:	**kmilkman@wharton.upenn.edu**
HS Training Completed:	**Yes**
Training Expiration Date:	**07/29/2015**
Name of course completed :	**CITI Protection of Human Subjects Research Training - ORA**

Study Contacts

Name:	**PENG, LILI**
Dept / School / Div:	**8327 - Regulatory Affairs**
Campus Address Mail Code	
Address:	**17177 HANOVER LANE**
City State Zip:	**EDEN PRAIRIE MN 553470000**
Phone:	
Fax:	
Pager:	
Email:	**penglili@wharton.upenn.edu**
HS Training Completed:	**Yes**
Training Expiration Date:	**09/08/2016**
Name of course completed :	**CITI Protection of Human Subjects Research Training - ORA**

Other Investigator

Name:	**TUCKFIELD, BRADFORD J**
Dept / School / Div:	**8327 - Regulatory Affairs**
Campus Address<br>Mail Code	
Address:	**11712 UPLANDS RIDGE DR.**
City State Zip:	**AUSTIN TX 787380000**
Phone:	
Fax:	
Pager:	
Email:	**brt@wharton.upenn.edu**
HS Training Completed:	**Yes**
Training Expiration Date:	**01/21/2016**
Name of course completed :	**CITI Protection of Human Subjects Research Training - ORA**

Responsible Org (Department/School/Division):

709 - Operations, Information and Decisions

Key Study Personnel

None

Disclosure of Significant Financial Interests*
Does any person who is responsible for the design, conduct, or reporting of this research protocol have a **FINANCIAL INTEREST**?
No

Penn Intellectual Property*
To the best of the Principal Investigator's knowledge, does this protocol involve the testing, development or evaluation of a drug, device, product, or other type of intellectual property (IP) that is owned by or assigned to the University of Pennsylvania?

Certification
I have reviewed the *Financial Disclosure and Presumptively Prohibited Conflicts for Faculty Participating in Clinical Trials* and the *Financial Disclosure Policy for Research and Sponsored Projects* with all persons who are responsible for the design, conduct, or reporting of this research; and all required Disclosures have been attached to this application.
Yes

Social and Biological Sciences

Study Instruments
Discuss the particulars of the research instruments, questionnaires and other evaluation instruments in detail. Provide validation documentation and or procedures to be used to validate instruments. For well know and generally accepted test instruments the detail here can be brief. More detail may be required for a novel or new instrument. For ethnographic studies identify any study instruments to be used (i.e. for deception studies) and describe in detail where, when and how the study will be conducted and who or what are the subjects of study. Note: For more information on how to conduct ethical and valid ethnographic research, follow the link For oral histories or interviews provide the general framework for questioning and means of data collection. If interviews or groups settings are to be audio taped or video taped describe in detail the conditions under which it will take place. Include a copy of any novel

or new test instruments with the IRB submission.
The experiment will utilize survey instruments and questionnaires in order to assess the progress of subjects' goal achievements or habit making. These will be sent to participants at regular intervals throughout the experiment, and a final survey and feedback form will also be used at the conclusion of the survey. Additionally, AchieveMint uses a feedback system of reward points for its users, and these reward points may also be tracked in order to evaluate the progress of users' goals or habits.

Group Modifications
Describe necessary changes that will or have been made to the study instruments for different groups.
Any modifications for different groups will be minor. All groups will be evaluated using essentially the same set of survey instruments, but the wording within surveys may be modified slightly to be appropriate for group sets.

Method for Assigning Subjects to Groups
Describe how subjects will be randomized to groups.
In field experiments, subjects will be randomized into a control and a treatment group. Both AchieveMint and our independent study may provide a list of identifiers for participants. For studies implemented by us, participants will be randomly assignment to conditions. For studies implemented by AchieveMint, participants will be randomly assigned to conditions without my direct involvement.

Administration of Surveys and/or Process
Describe the approximate time and frequency for administering surveys and/or evaluations. For surveys, questionnaires and evaluations presented to groups and in settings such as high schools, focus group sessions or community treatment centers explain how the process will be administered and who will oversee the process. For instance, discuss the potential issues of having teachers and other school personnel administer instruments to minors who are students especially if the content is sensitive in nature. Describe the procedure for audio and videotaping individual interviews and/or focus groups and the storage of the tapes. For instance, if audio tape recording is to be used in a classroom setting, describe how this will be managed if individuals in the class are not participating in the study. Explain if the research involves the review of records (including public databases or registries) with identifiable private information. If so, describe the type of information gathered from the records and if identifiers will be collected and retained with the data after it is retrieved. Describe the kinds of identifiers to be obtained, (i.e. names, social security numbers) and how long the identifiers will be retained and justification for use.
Studies will have several surveys or questionnaires that are distributed to participants via mail, email, or direct elicitation.

Data Management
Describe how and who manages confidential data, including how and where it will be stored and analyzed. For instance, describe if paper or electronic report forms will be used, how corrections to the report form will be made, how data will be entered into any database, and the person(s) responsible for creating and maintaining the research database. Describe the use of pseudonyms, code numbers and how listing of such identifiers will be kept separate from the research data.
AchieveMint will collect the data in the course of its normal business operations and the normal functionality of its application. All information will be unidentifiable, and data collected will be kept in a secure hard-drive that is password protected.

Radiation Exposure*
Are research subjects receiving any radiation exposure (e.g. X-rays, CT, Fluoroscopy, DEXA, pQCT, FDG, Tc-99m, etc.) that they would not receive if they were not enrolled in this protocol?
No

Human Source Material*
Does this research include collection or use of human source material (i.e., human blood, blood products, tissues or body fluids)?
No

CACTIS and CT Studies*
Does the research involve Center for Advanced Computed Tomography Imaging Services (CACTIS) and CT studies that research subjects would not receive if they were not part of this protocol?
No

CAMRIS and MRI Studies*
Does the research involve Center for Advanced Magnetic Resonance Imaging and Spectroscopy (CAMRIS) and MRI studies that research subjects would not receive if they were not part of this protocol?
No

Cancer Related research not being conducted by an NCI cooperative group*
Does this protocol involve cancer-related studies in any of the following categories?
No

Medical Information Disclosure*
Does the research proposal involve the use and disclosure of research subject's medical information for research purposes?
No

CTRC Resources*
Does the research involve CTRC resources?
No

If the answer is YES, indicate which items is is provided with this submission:

Use of UPHS services*
Does your study require the use of University of Pennsylvania Health System (UPHS) services, tests or procedures*, whether considered routine care or strictly for research purposes?
No

Primary Focus*
Sociobehavioral (i.e. observational or interventional)

Protocol Interventions

	**Sociobehavioral (i.e. cognitive or behavioral therapy)**
	**Drug**
	**Device - therapeutic**
	**Device - diagnostic (assessing a device for sensitivity or specificity in disease diagnosis)**
	**Surgical**
	**Diagnostic test/procedure (research-related diagnostic test or procedure)**
	**Obtaining human tissue for basic research or biospecimen bank**
x	**Survey instrument**
	**None of the above**

The following documents are currently attached to this item:

There are no documents attached for this item.

Department budget code

None

Multi-Site Research

Other Sites

No other sites

Management of Information for Multi-Center Research
We will share information between Achievemint and Penn by uploading files to a secure server to which both parties have access.

The following documents are currently attached to this item:

There are no documents attached for this item.

Protocol

Abstract
The goal of this set of projects is to apply insights from behavioral economics literature and theory to help people accomplish personal goals and form good personal habits. We will be working with a company called AchieveMint, a company that has developed an application and software process to encourage users' healthy activities and develop insights into their routines. This research is broadly applicable, as everyone has goals they wish to achieve and personal habits whose improvements could better their health and personal lives. The set of experiments we plan to run with AchieveMint will all be focused on discovering what factors enable and encourage users to achieve goals (or why they fail), and what factors enable and encourage users to start new healthy habits or give up bad habits.

Objectives

Overall objectives
The objective is to test various hypotheses about goal achievement and habit forming that are based in behavioral economics theory and literature. We will be experiments with AchieveMint and develop and test hypotheses about various aspects of how users achieve their goals and develop healthy habits.

Primary outcome variable(s)
The primary outcome variables are whether the users achieves his/her goal or succeeds in forming/breaking his intended habit.

Secondary outcome variable(s)
Secondary outcome variables include those such as timing and pace of goal achievement, and user feedback during the process and at completion.

Background
Previous research has highlighted the value of applying insights from behavioral economics to persuade people to make better decisions, and this work builds on those ideas (Thaler and Sunstein, 2008).

Study Design

Phase*
Not applicable

Design
We will be analyzing data from a set of randomized, controlled experiments that will be implemented by our partner firm AchieveMint. AchieveMint focuses on encouraging users to achieve their personal goals and rewards users by a rewards point system. For our studies, users will either volunteer to be part of the study and then randomly assigned to different treatments, or users with similar goals will be recruited for a larger pool and then randomly assigned to treatments.

Study duration
Participation in the studies varies depending on both the length the user has personally assigned to achieve his/her goal, or the study length incorporated as a part of our experimental design. Typically, studies start to finish should take several weeks, which is enough time to enable people to achieve their goals or form/break habits.

Resources necessary for human research protection
Describe research staff and justify that the staff are adequate in number and qualifications to conduct the research. Describe how you will ensure that all staff assisting with the research are adequately

informed about the protocol and their research related duties. Please allow adequate time for the researchers to conduct and complete the research. Please confirm that there are adequate facilities for the research.
The staff at AchieveMint will conduct the study along with myself and my principal investigators (listed previously in the application). Myself and my investigators have completed CITI training and discussed the approriate protocols for safe human subjects research with the staff at AchieveMint. The population that we have access to for this research is the University of Pennsylvania community and the users of AchieveMint's application.

Characteristics of the Study Population

Target population
AchieveMint users and University of Pennsylvania community members who have a personal goal they would like to achieve or a personal habit that they would like to change.

Subjects enrolled by Penn Researchers
0

Subjects enrolled by Collaborating Researchers
0

Accrual
Through our collaboration with AchieveMint, we will have access to the entire user base of their application.. Various experiments will be targeted at subsets of the subject population, with the goal of there being enough participants per study to enable results to be statistically significant.

Key inclusion criteria
AchieveMint's users base generally range in age 18 to 100 and are both male and female.

Key exclusion criteria
No children, prisoners, or other vulnerable subjects will be recruited. Those who choose not to participate in the study will also not be included.

Vulnerable Populations

- **Children Form**
- **Pregnant women (if the study procedures may affect the condition of the pregnant woman or fetus) Form**
- **Fetuses and/or Neonates Form**
- **Prisoners Form**
- **Other**
- x **None of the above populations are included in the research study**

The following documents are currently attached to this item:

There are no documents attached for this item.

Populations vulnerable to undue influence or coercion
Not applicable

Subject recruitment
Subjects may be recruited from the Achievemint population base based on their particular goal. For example, AchieveMint users who want to start a diet may be recruited to be part of a voluntary study.

Will the recruitment plan propose to use any Penn media services (communications, marketing, etc.) for outreach via social media avenues (examples include: Facebook, Twitter, blogging, texting, etc.) or does the study team plan to directly use social media to recruit for the research?
No

The following documents are currently attached to this item:

There are no documents attached for this item.

Subject compensation*
Will subjects be financially compensated for their participation?
Yes

The following documents are currently attached to this item:

There are no documents attached for this item.

If there is subject compensation, provide the schedule for compensation per study visit or session and total amount for entire participation, either as text or separate document

The AchieveMint application offers users small financial compensation as part of its normal business operations and independent of our study design and without our involvement.

Study Procedures

Suicidal Ideation and Behavior
Does this research qualify as a clinical investigation that will utilize a test article (ie- drug or biological) which may carry a potential for central nervous system (CNS) effect(s)?
No

Procedures
Subjects with the same goal using the AchieveMint platform will be recruited. For example, AchieveMint users who want to start a diet. Evaluation and information that is goal specific will be collected from the voluntary participants before the study. For example, a weigh-in for participants who want to start a diet, or a preliminary food log, before they start the study. Throughout the length of the study, participants will be contacted at regular intervals (of which they will be cognizant of prior to the study), and information about the progress of their goal will be collected. In this example, participants might be emailed twice weekly and asked to fill out a questionnaire of the things they've done in the past few days to try to eat healthy food. The surveys and questionnaires will be designed specifically to collect data about the outcome variables, with which the success of the user in their goal will be measured. After the study concludes, participants will be asked for a final exit questionnaire, which will assess the extent to which they were successful and how they felt about their goal progress and success. The data collected will be analyzed using statistical significance tests and regression analyses, as well as other goal-specific measures (in this case, weight lost or other health measures). We will contact subjects via the communication channels established by Achievemint. These include email (Achievemint has addresses on file) and in-app messages (for voluntary site visitors). Some interventions will involve announcing patterns of incentive bonuses to users via email. For example, a user may be told that they will receive double points today, and triple points tomorrow, or triple points today, and double points tomorrow. See information in the attached Appendix A for more information about this intervention.

The following documents are currently attached to this item:

There are no documents attached for this item.

Deception
Does your project use deception?
No

Analysis Plan
Statistical tests of significance and regression analyses will be used to evaluate the user data between conditions.

The following documents are currently attached to this item:

There are no documents attached for this item.

Are you conducting research outside of the United States?
No

Data confidentiality

- x **Paper-based records will be kept in a secure location and only be accessible to personnel involved in the study.**
- x **Computer-based files will only be made available to personnel involved in the study through the use of access privileges and passwords.**
- **Prior to access to any study-related information, personnel will be required to sign statements agreeing to protect the security and confidentiality of identifiable information.**
- x **Wherever feasible, identifiers will be removed from study-related information.**
- **A Certificate of Confidentiality will be obtained, because the research could place the subject at risk of criminal or civil liability or cause damage to the subject's financial standing, employability, or liability.**
- x **A waiver of documentation of consent is being requested, because the only link between the subject and the study would be the consent document and the primary risk is a breach of confidentiality. (This is not an option for FDA-regulated research.)**
- x **Precautions are in place to ensure the data is secure by using passwords and encryption, because the research involves web-based surveys.**
- **Audio and/or video recordings will be transcribed and then destroyed to eliminate audible identification of subjects.**

Subject Confidentiality
No identifying information about the subjects will be collected. I will not make the data collected in this study publicly available. I will keep the data on a personal laptop and hard-drive, which will be password-protected and contain firewall-protected servers to ensure that only I have access to it.

Sensitive Research Information*
Does this research involve collection of sensitive information about the subjects that should be excluded from the electronic medical record?
No

Subject Privacy
Privacy refers to the person's desire to control access of others to themselves. Privacy concerns people, whereas confidentiality concerns data. Describe the strategies to protect privacy giving consideration to the following: The degree to which privacy can be expected in the proposed research and the safeguards that will be put into place to respect those boundaries. The methods used to identify and contact potential participants. The settings in which an individual will be interacting with an investigator. The privacy guidelines developed by relevant professions, professional associations and scholarly disciplines (e.g., psychiatry, genetic counseling, oral history, anthropology, psychology).
Only AchieveMint users - who have voluntarily signed up for the service and are already using the AchieveMint platform - and University of Pennsylvania members will be contacted. All contact will be relatively innocuous and conducted through direct mail or email.

Data Disclosure
Will the data be disclosed to anyone who is not listed under Personnel?
Data will not be disclosed to anyone who is not listed under Personnel.

*Data Protection**

- **Name**
- **Street address, city, county, precinct, zip code, and equivalent geocodes**
- **All elements of dates (except year) for dates directly related to an individual and all ages over 89**
- **Telephone and fax number**
- **Electronic mail addresses**
- **Social security numbers**
- **Medical record numbers**
- **Health plan ID numbers**
- **Account numbers**
- **Certificate/license numbers**
- **Vehicle identifiers and serial numbers, including license plate numbers**
- **Device identifiers/serial numbers**
- **Web addresses (URLs)**
- **Internet IP addresses**
- **Biometric identifiers, incl. finger and voice prints**
- **Full face photographic images and any comparable images**
- **Any other unique identifying number, characteristic, or code**
- x **None**

Does your research request both a waiver of HIPAA authorization for collection of patient information and involve providing Protected Health Information ("PHI") that is classified as a "limited data set" (city/town/state/zip code, dates except year, ages less than 90 or aggregate report for over 90) to a recipient outside of the University of Pennsylvania covered entity?
No

Tissue Specimens Obtained as Part of Research*
Are Tissue Specimens being obtained for research?
No

Tissue Specimens - Collected during regular care*
Will tissue specimens be collected during regulator clinical care (for treatment or diagnosis)?
No

Tissue Specimens - otherwise discarded*
Would specimens otherwise be discarded?
No

Tissue Specimens - publicly available*
Will tissue specimens be publicly available?
No

Tissue Specimens - Collected as part of research protocol*
Will tissue specimens be collected as part of the research protocol?
No

Tissue Specimens - Banking of blood, tissue etc. for future use*
Does research involve banking of blood, tissue, etc. for future use?
No

Genetic testing
If genetic testing is involved, describe the nature of the tests, including if the testing is predicative or exploratory in nature. If predictive, please describe plan for disclosing results to subjects and provision of genetic counseling. Describe how subject confidentiality will be protected Note: If no genetic testing is to be obtained, write: "Not applicable."
Not applicable.

Consent

1. Consent Process

Overview
I am requesting a waiver of informed consent (please see below).

Children and Adolescents
Not applicable.

Adult Subjects Not Competent to Give Consent
Not applicable.

2. Waiver of Consent

Waiver or Alteration of Informed Consent*
Waiver of written documentation of informed consent: the research presents no more than minimal risk of harm to subjects and involves no procedures for which written consent is normally required outside of the research context

Minimal Risk*

Impact on Subject Rights and Welfare*

Waiver Essential to Research*

Additional Information to Subjects

Written Statement of Research*
No

If no written statement will be provided, please provide justificiation

Participants have voluntarily participated in the services provided by the AchieveMint application outside of the scope of this research. Because the data used in these studies are collected and sourced as part of normal business operations of AchieveMint, I am seeking a waiver of the requirement to obtain informed consent for participation in this research. As described above, field experiment subjects are voluntary users for the AchieveMint interface. HHS regulation 46.116 provides that an IRB may waive the requirements to obtain informed consent when "1) The research involves no more than minimal risk to the subjects; 2) The waiver or alteration will not adversely affect the rights and welfare of the subjects; 3) The research could not practicably be carried out without the waiver or alteration; 4) Whenever appropriate, the subjects will be provided with additional pertinent information after participation." I believe that the risks to the subjects are no more than minimal. (1) I will not collect any identifying information about the subjects. I will not make the data collected in these studies publicly available. I will keep the data on my personal laptop or password- and firewall-protected servers to ensure that only I have access to it and in a locked cabinet. (2) These studies pose minimal risks to health or well-being. The AchieveMint application is a service that consumers voluntarily consume in order to help them achieve a goal or a break a habit, and AchieveMint incentivizes its users to better their personal lives. The types of behaviors users engage in during the course of their use of AchieveMint are entirely under their control, and the benefits of working to achieve personal goals encouraged by the AchieveMint are believed to substantially outweigh the risks. I believe I have

established in the previous sections that the risks to the subjects are no more than minimal and that the rights and welfare of the subjects will not be adversely affected by the studies proposed herein. Subjects are free to seek or forego the goals they've set in AchieveMint and have full discretion over the behaviors they engaged in to try to achieve the goal they've set for themselves. After the study, subjects remain free to both use the AchieveMint to achieve their personal goals and habits as they want to and work towards achieving a personal goal. In addition, the interventions in these studies, which are designed with the goal of helping users gain insight into the efficacy of their goal progress, should presumably benefit subjects welfare. Additionally, it is not feasible to obtain informed consent from subjects in these studies. It would be logistically difficult to obtain informed consent from subjects before they intend to use AchieveMint, and the set of studies essentially provides optional alterations to services and insights to services that users voluntarily agree to consume. Consumers presumably have informed themselves of how the AchieveMint service works, what it intends to do, and how it will benefit them. Essentially, users have engaged a personal cost-benefit analysis before signing up to use the AchieveMint that the personal benefit of using the AchieveMint to help them achieve their goal outweighs any marginal monetary cost or personal inconvenience. AchieveMint users will know about the service, and no particular behaviors will be forced onto them through the study. The Achievemint user agreement includes a stipulation that their information may be used by third parties to investigate how to improve their services. So, Achievemint users should be aware of this. In conclusion, given that this research and my involvement as a researcher poses minimal risk and doesn't adversely affect the rights and welfare of participants, and given that the subject population as a whole could benefit if the studies generate generalizable knowledge, I request that requirements to obtain informed consent from each user be waived.

The following documents are currently attached to this item:

There are no documents attached for this item.

Risk / Benefit

Potential Study Risks
No identifying information about the subjects will be collected, and the data collected in the studies will not be publicly available. The data will be kept on a personal laptop or password encrypted hard drive to limit and control access to it. The studies pose minimal risks to healthy or well-being. The types of data and activities to be studied are those that users of AchieveMint voluntarily agree to by signing up for the service, and are part of AchieveMint's normal business operations. The questionnaires given to participants are sometimes annoying, but participant is voluntary and participants are compensated for their time.

Potential Study Benefits
Potential benefits to be gained by the individual subject are those relating to the emotional and physical satisfaction of achieving a goal or breaking a bad habit, as well as the positive effects on that subject's physical well-being. Understanding the factors that lead to goal achievement and habit formation will have positive benefits to society because the research findings may generalize to help people achieve their goals and break unhealthy habits.

Alternatives to Participation (optional)

Data and Safety Monitoring
The PI will monitor the data safety in this study. I will not collect any identifying information about the subjects. I will not make the data collected in this study publicly available. I will keep the data on my personal laptop or password- and firewall-protected servers to ensure that only I have access to it. Participants shouldn't be exposed to any realizable safety concerns. The studies alters services that participants have voluntarily chosen to consume, so participants are already aware of potential personal safety concerns that may arise during their use. No user names will be collected, which will maintain privacy. Data integrity will be assessed through iterative evaluations of the data and data analysis.

The following documents are currently attached to this item:

There are no documents attached for this item.

Risk / Benefit Assessment
The reward to risk ratio is very high. The study seeks to benefit those who have personal goals they'd like to achieve, and there is minimal risk involved in participation. I believe the potential benefits to this research (finding more effective ways to achieve goals) substantially outweigh the risks.

General Attachments

The following documents are currently attached to this item:

Cover Letter (coverletter.docx)

Additional forms (appendixa.docx)

Cover Letter (coverletter.docx)

Questionnaires (instrument.docx)

Questionnaires (questionnaire.docx)

Cover Letter (coverletter.docx)
