# Supplementary material for: Effect of Different Financial Incentive Structures on Promoting Physical Activity Among Adults: A Randomized Clinical Trial
Source: JAMA Netw Open. 2019 Aug 23;2(8):e199863. doi: 10.1001/jamanetworkopen.2019.9863 (PMC6714021; doi:10.1001/jamanetworkopen.2019.9863)
Supplement: Supplement 2. — eTable. Schedule of Incentive Rates Offered per Step on Each of 14 Days of Intervention by Experimental Condition eFigure 1. Program Announcement Email for the Control Condition eFigure 2. Program Announcement Email for the Constant Incentive Condition eFigure 3. Program Announcement Email for the Increasing Incentive Condition eFigure 4. Program Announcement Email for the Decreasing Incentive Condition eFigure 5. Reminder Email for the Control Condition eFigure 6. Reminder Email for the Treatment Conditions [file jamanetwopen-2-e199863-s002.pdf]

Supplementary Online Content

Bachireddy C, Joung A, John LK, et al. Effect of different financial incentive structures on promoting physical activity among adults: a randomized clinical trial. *JAMA Netw Open.* 2019;2(8):e199863. doi:10.1001/jamanetworkopen.2019.9863

eTable. Schedule of Incentive Rates Offered per Step on Each of 14 Days of Intervention by Experimental Condition
eFigure 1. Program Announcement Email for the Control Condition
eFigure 2. Program Announcement Email for the Constant Incentive Condition
eFigure 3. Program Announcement Email for the Increasing Incentive Condition
eFigure 4. Program Announcement Email for the Decreasing Incentive Condition
eFigure 5. Reminder Email for the Control Condition
eFigure 6. Reminder Email for the Treatment Conditions

This supplementary material has been provided by the authors to give readers additional information about their work.

eTable. Schedule of Incentive Rates Offered per Step on Each of 14 Days of Intervention by Experimental Condition

Schedule of Incentives	Control	Constant	Increasing	Decreasing
Day 1	$0.00001	$0.00020	$0.00005	$0.00035
Day 2	$0.00001	$0.00020	$0.00005	$0.00035
Day 3	$0.00001	$0.00020	$0.00010	$0.00030
Day 4	$0.00001	$0.00020	$0.00010	$0.00030
Day 5	$0.00001	$0.00020	$0.00015	$0.00025
Day 6	$0.00001	$0.00020	$0.00015	$0.00025
Day 7	$0.00001	$0.00020	$0.00020	$0.00020
Day 8	$0.00001	$0.00020	$0.00020	$0.00020
Day 9	$0.00001	$0.00020	$0.00025	$0.00015
Day 10	$0.00001	$0.00020	$0.00025	$0.00015
Day 11	$0.00001	$0.00020	$0.00030	$0.00010
Day 12	$0.00001	$0.00020	$0.00030	$0.00010
Day 13	$0.00001	$0.00020	$0.00035	$0.00005
Day 14	$0.00001	$0.00020	$0.00035	$0.00005

© 2019 Bachireddy C et al. *JAMA Network Open.*

eFigure 1. Program Announcement Email for the Control Condition

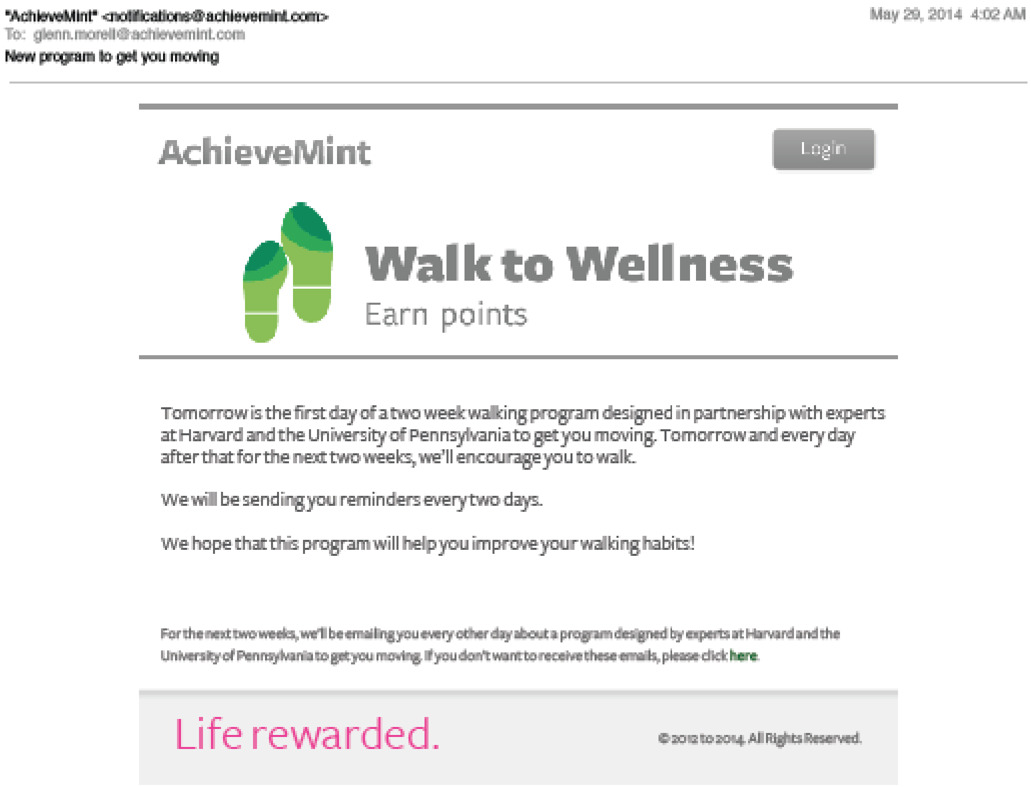

"AchieveMint" <notifications@achievemint.com>
To: glenn.morell@achievemint.com
New program to get you moving

May 29, 2014 4:02 AM

AchieveMint

Login

Walk to Wellness

Earn points

Tomorrow is the first day of a two week walking program designed in partnership with experts at Harvard and the University of Pennsylvania to get you moving. Tomorrow and every day after that for the next two weeks, we'll encourage you to walk.

We will be sending you reminders every two days.

We hope that this program will help you improve your walking habits!

For the next two weeks, we'll be emailing you every other day about a program designed by experts at Harvard and the University of Pennsylvania to get you moving. If you don't want to receive these emails, please click here.

Life rewarded.

©2012 to 2014. All Rights Reserved.

eFigure 2. Program Announcement Email for the Constant Incentive Condition

"AchieveMint" <notifications@achievemint.com> May 29, 2014 4:02 AM
To: glenn.morell@achievemint.com
New program to get you moving (with bonus points)

AchieveMint Login

Walk to Wellness
Earn points

Tomorrow is the first day of a two week walking program designed in partnership with experts at Harvard and the University of Pennsylvania to get you moving. Tomorrow and every day after that for the next two weeks, we'll encourage you to walk by multiplying the points you earn for walking by 20.

Specifically, your bonuses from AchieveMint over the next two weeks will follow this schedule:

June 2014

Sunday	Monday	Tuesday	Wednesday	Thursday	Friday	Saturday
1	2 20x	3 20x	4 20x	5 20x	6 20x	7 20x
8 20x	9 20x	10 20x	11 20x	12 20x	13 20x	14 20x
15 20x	16	17	18	19	20	21
22	23	24	25	26	27	28
29	30					

We will be sending you reminders every two days about upcoming bonuses.

We hope that this program will help you improve your walking habits!

For the next two weeks, we'll be emailing you every other day about a program designed by experts at Harvard and the University of Pennsylvania to get you moving. If you don't want to receive these emails, please click here.

© 2019 Bachireddy C et al. *JAMA Network Open.*

eFigure 3. Program Announcement Email for the Increasing Incentive Condition

"AchieveMint" <notifications@achievemint.com>
To: glenn.morell@achievemint.com
New program to get you moving (with bonus points)

May 29, 2014 4:02 AM

AchieveMint

Login

Walk to Wellness

Earn points

Tomorrow is the first day of a two week walking program designed in partnership with experts at Harvard and the University of Pennsylvania to get you moving. Tomorrow and the day after that, we'll encourage you to walk by multiplying the points you earn for walking by 5. After that (i.e., in 3 days' time), we'll multiply the points you earn for walking by 10, and then two days later, your bonus multiplier will increase again. Specifically, your multiplier will increase by 5 every two days, up to a multiplier of 35 on the last two days of this program.

Specifically, your bonuses from AchieveMint over the next two weeks will follow this schedule:

June 2014

Sunday	Monday	Tuesday	Wednesday	Thursday	Friday	Saturday
1	2 5x	3 5x	4 10x	5 10x	6 15x	7 15x
8 20x	9 20x	10 25x	11 25x	12 30x	13 30x	14 35x
15 35x	16	17	18	19	20	21
22	23	24	25	26	27	28
29	30					

We will be sending you reminders every two days about upcoming bonuses.

We hope that this program will help you improve your walking habits!

For the next two weeks, we'll be emailing you every other day about a program designed by experts at Harvard and the University of Pennsylvania to get you moving. If you don't want to receive these emails, please click here.

© 2019 Bachireddy C et al. *JAMA Network Open.*

eFigure 4. Program Announcement Email for the Decreasing Incentive Condition

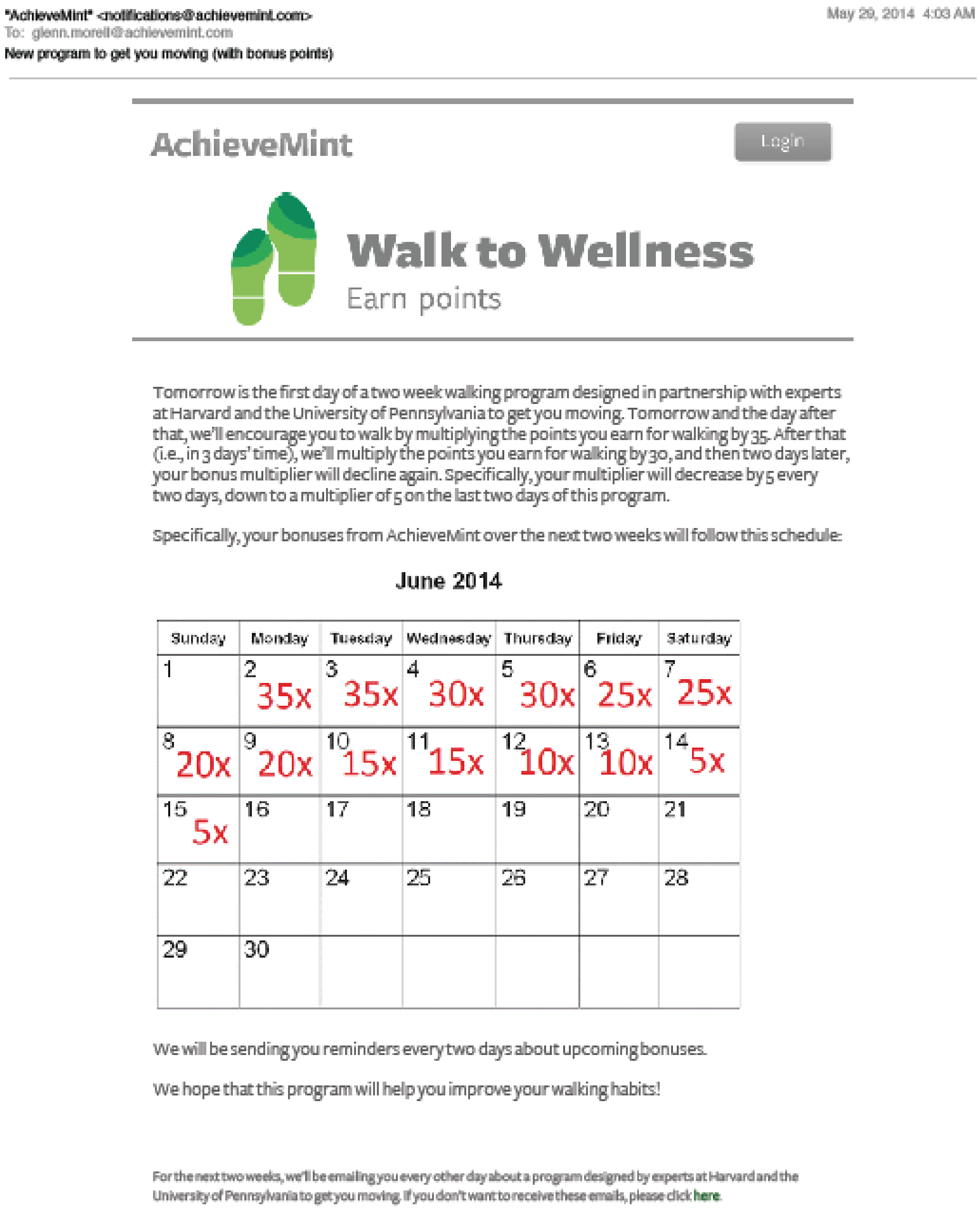

"AchieveMint" <notifications@achievemint.com>
To: glenn.morell@achievemint.com
New program to get you moving (with bonus points)

May 29, 2014 4:03 AM

AchieveMint

Login

Walk to Wellness

Earn points

Tomorrow is the first day of a two week walking program designed in partnership with experts at Harvard and the University of Pennsylvania to get you moving. Tomorrow and the day after that, we'll encourage you to walk by multiplying the points you earn for walking by 35. After that (i.e., in 3 days' time), we'll multiply the points you earn for walking by 30, and then two days later, your bonus multiplier will decline again. Specifically, your multiplier will decrease by 5 every two days, down to a multiplier of 5 on the last two days of this program.

Specifically, your bonuses from AchieveMint over the next two weeks will follow this schedule:

June 2014

Sunday	Monday	Tuesday	Wednesday	Thursday	Friday	Saturday
1	2 35x	3 35x	4 30x	5 30x	6 25x	7 25x
8 20x	9 20x	10 15x	11 15x	12 10x	13 10x	14 5x
15 5x	16	17	18	19	20	21
22	23	24	25	26	27	28
29	30					

We will be sending you reminders every two days about upcoming bonuses.

We hope that this program will help you improve your walking habits!

For the next two weeks, we'll be emailing you every other day about a program designed by experts at Harvard and the University of Pennsylvania to get you moving. If you don't want to receive these emails, please click here.

© 2019 Bachireddy C et al. *JAMA Network Open.*

eFigure 5. Reminder Email for the Control Condition

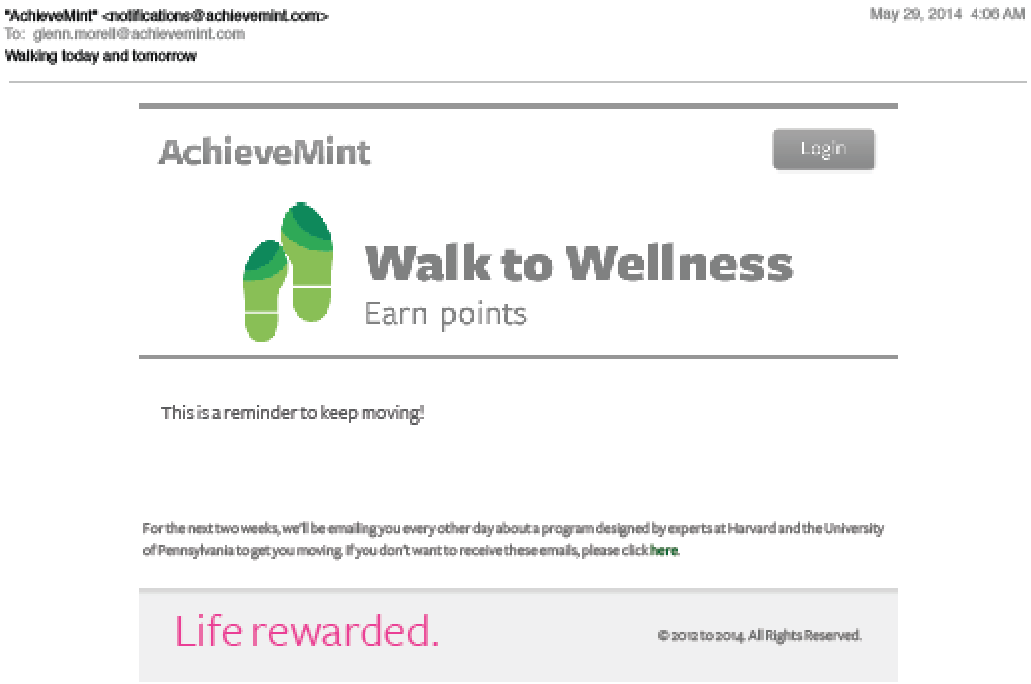

© 2019 Bachireddy C et al. *JAMA Network Open.*

eFigure 6. Reminder Email for the Treatment Conditions (Information About the Extra Points Promised Varied by Treatment Condition and Day of Intervention)

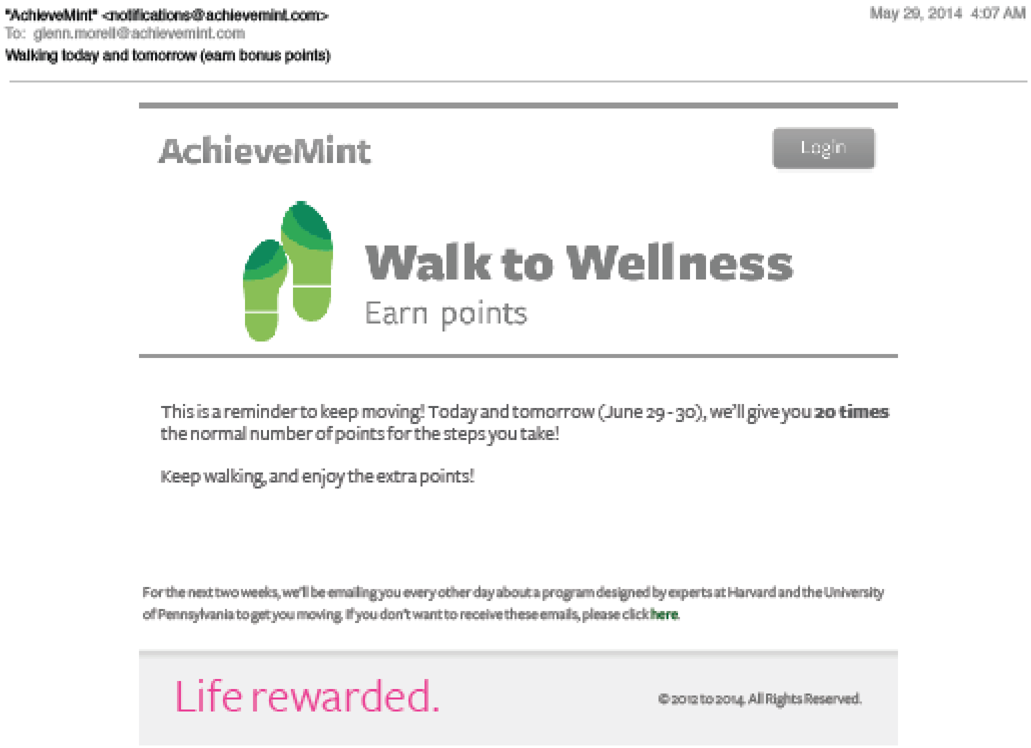

"AchieveMint" <notifications@achievemint.com>
To: glenn.morell@achievemint.com
Walking today and tomorrow (earn bonus points)

May 29, 2014 4:07 AM

AchieveMint

Login

Walk to Wellness

Earn points

This is a reminder to keep moving! Today and tomorrow (June 29 - 30), we'll give you **20 times** the normal number of points for the steps you take!

Keep walking, and enjoy the extra points!

For the next two weeks, we'll be emailing you every other day about a program designed by experts at Harvard and the University of Pennsylvania to get you moving. If you don't want to receive these emails, please click **here**.

Life rewarded.

©2012 to 2014. All Rights Reserved.
