# Supplementary material for: Effect of Different Financial Incentive Structures on Promoting Physical Activity Among Adults: A Randomized Clinical Trial
Source: JAMA Netw Open. 2019 Aug 23;2(8):e199863. doi: 10.1001/jamanetworkopen.2019.9863 (PMC6714021; doi:10.1001/jamanetworkopen.2019.9863)
Supplement: Supplement 3. — Data Sharing Statement [file jamanetwopen-2-e199863-s003.pdf]

# Data Sharing Statement

Bachireddy. Effect of Different Financial Incentive Structures on Promoting Physical Activity Among Adults. *JAMA Netw Open*. Published August 23, 2019. 10.1001/jamanetworkopen.2019.9863

**Data**
**Data available:** No

**Additional Information**
**Explanation for why data not available:** The collected data for this study contains personally identifying information that poses a risk of re-identification for study participants. As part of our efforts to ensure the welfare of participants and maintain their privacy, we have committed to the data provider that we will share data through a Data Use Agreement that asks third party researchers to 1) request the data explicitly from the authors: 2) pledge to not attempt participant re-identification; 3) establish safeguards for participant privacy; and 4) bear the liability of leaking the data in the public domain.
